# Evaluation of calibrated and uncalibrated optical imaging approaches for relative cerebral oxygen metabolism measurements in awake mice

**DOI:** 10.1088/1361-6579/ad3a2d

**Published:** 2024-04-24

**Authors:** A E Toader, M Fukuda, A L Vazquez

**Affiliations:** 1 Departments of Radiology, University of Pittsburgh, Pittsburgh PA 15217, United States of America; 2 Bioengineering, University of Pittsburgh, Pittsburgh PA 15217, United States of America

**Keywords:** CMRO_2_, calibration, hypercapnia, optical imaging

## Abstract

*Objective*. The continuous delivery of oxygen is critical to sustain brain function, and therefore, measuring brain oxygen consumption can provide vital physiological insight. In this work, we examine the impact of calibration and cerebral blood flow (CBF) measurements on the computation of the relative changes in the cerebral metabolic rate of oxygen consumption (rCMRO_2_) from hemoglobin-sensitive intrinsic optical imaging data. Using these data, we calculate rCMRO_2_, and calibrate the model using an isometabolic stimulus. *Approach*. We used awake head-fixed rodents to obtain hemoglobin-sensitive optical imaging data to test different calibrated and uncalibrated rCMRO_2_ models. Hypercapnia was used for calibration and whisker stimulation was used to test the impact of calibration. *Main results*. We found that typical uncalibrated models can provide reasonable estimates of rCMRO_2_ with differences as small as 7%–9% compared to their calibrated models. However, calibrated models showed lower variability and less dependence on baseline hemoglobin concentrations. Lastly, we found that supplying the model with measurements of CBF significantly reduced error and variability in rCMRO_2_ change calculations. *Significance*. The effect of calibration on rCMRO_2_ calculations remains understudied, and we systematically evaluated different rCMRO_2_ calculation scenarios that consider including different measurement combinations. This study provides a quantitative comparison of these scenarios to evaluate trade-offs that can be vital to the design of blood oxygenation sensitive imaging experiments for rCMRO_2_ calculation.

## Introduction

The continuous delivery of oxygen is critical to sustain brain function. Oxygen is delivered to tissue by arterial blood and most of the exchange occurs at the capillary level. Fick’s principle is used to describe oxygen delivery and consumption, where delivery depends on cerebral blood flow (CBF) and, in its common form, is in steady balance with its consumption in tissue because there is no net storage of oxygen. Direct methods to measure oxygen consumption in the brain have relied on measurements of tissue oxygen tension using polarographic oxygen microelectrodes or residual oxygen using positron emission tomography (PET) (Mintun *et al* 1984, Piilgaard and Lauritzen 2009). Although these methods are directly sensitive to oxygen, they still require measurements of oxygen delivery in order to calculate consumption. Other more widely used methods are sensitive to the hemoglobin oxidative state which are used to calculate cerebral metabolic rate of oxygen consumption (CMRO_2_) based on changes in the blood oxygenation level (Davis *et al* 1998, Hoge *et al* 1999a, Culver *et al* 2003, Dunn *et al* 2005, Huppert *et al* 2007, Piilgaard and Lauritzen 2009, Lin *et al* 2013, Verdecchia *et al* 2013, Kainerstorfer *et al* 2014, Takuwa *et al* 2014, Yucel *et al* 2014, Barrett and Suresh 2015, Baker *et al* 2019, Dahlqvist *et al* 2020, Ko *et al* 2020, Saetra *et al* 2020, Acharya *et al* 2022, Chong *et al* 2015, 2022). Hemoglobin is the main oxygen carrier in blood and has magnetic properties, allowing MRI sensitivity (Kim 2018). It is also a strong absorber of light, and its absorption properties depend on the light wavelength as well as its oxygen saturation (i.e. the number of oxygen molecules bound to it). Hence, optical imaging methods can be used similarly to MRI to measure changes in the oxygenation level of blood as it traverses through brain tissue. These measurements can then be used to calculate oxygen consumption in a brain region with good spatial and temporal resolution.

To make quantitative measurements of CMRO2 from blood oxygenation measurements, a biophysical model that incorporates relevant physiology with changes in oxygen-sensitive signals is necessary. The physiological portion is based on Fick’s conservation of mass, which states that the amount of oxygen consumed in tissue is equal to the difference in arterial and venous oxygen (i.e. CMRO_2_ = CBF·(*C*_a_ − *C*_v_), where *C*_a_ and *C*_v_ denote the arterial and venous blood oxygen concentration), since there is no net tissue oxygen storage (Clanton *et al* 2013). The biophysical portion involves relating measured signals to the venous oxygen saturation since arterial blood is typically fully saturated with oxygen. Models have been developed for MRI signals (Davis *et al* 1998) as well as optical signals, the latter usually involving the modified Beer–Lambert Law (Dunn *et al* 2005). Since imaging signals do not generally yield absolute measurements, these models are simplified to quantify changes relative to baseline. Following this general approach, Hoge *et al* computed the changes in CMRO_2_ produced by visual stimuli in awake humans using calibrated fMRI and compared it to the same experiments using PET. The CMRO_2_ change computed by fMRI was 25 ± 4%, and using PET was 25 ± 5% (Hoge *et al* 1999a), indicating good agreement between PET and fMRI. Optical studies have reported similar changes in CMRO_2_ in response to sensory stimulation in animal studies, with increases between 5% and 10% to whisker stimulation in awake head-fixed mice and changes of about 10% to sensory stimulation in lightly sedated rats (Dunn *et al* 2005, Dahlqvist *et al* 2020).

An important aspect of this general approach is that an isometabolic stimulus is used to ‘calibrate’ the imaging signals to relative changes in CMRO_2_ (rCMRO_2_). This procedure is used to ensure the model’s physiological validity and calibrate it for changes in oxygen produced by changes in blood supply in the absence of changes in neural activity (Davis *et al* 1998, Hoge *et al* 1999b, Goodwin *et al* 2009, Lajoie *et al* 2017). Several groups have tested this assumption with electrophysiological recordings, and it has been determined to be reasonable for CO_2_ concentrations below 10% (Berwick *et al* 2005). Other calibration approaches have been proposed such as hyperoxia and breath-holding with some advantages and disadvantages, but hypercapnia remains the most common method (Kastrup *et al* 1999, Goodwin *et al* 2009). As a result, these methods have adopted names such as ‘Calibrated fMRI’ (Davis *et al* 1998, Yucel *et al* 2014, Lajoie *et al* 2017). However, this calibration step is not commonly used in optical studies (Culver *et al* 2003, Dunn *et al* 2005, Huppert *et al* 2007, Piilgaard and Lauritzen 2009, Lin *et al* 2013, Verdecchia *et al* 2013, Kainerstorfer *et al* 2014, Takuwa *et al* 2014, Barrett and Suresh 2015, Baker *et al* 2019, Dahlqvist *et al* 2020, Ko *et al* 2020, Saetra *et al* 2020, Acharya *et al* 2022, Chong *et al* 2015, 2022) because multi-wavelength optical data can be sufficient to calculate changes in oxygenation. Notwithstanding, various assumptions remain that impact CMRO_2_ calculation, such as known baseline concentrations for oxygenated and deoxygenated hemoglobin and their relative volume fractions.

In this work we evaluate calibrated and uncalibrated optical imaging approaches sensitive to blood oxygenation to measure relative changes in brain oxygen metabolism. To determine the reliability of these different methods to estimate CMRO_2_, we conducted two different types of experiments in partially overlapping mouse cohorts. In one group, we tested reliability at predicting no change in CMRO_2_ during hypercapnia gas administration, and in another group, we examined CMRO_2_ predictions to whisker stimulation. Lastly, the approaches we tested are sensitive to assumptions of the baseline oxygen extraction; hence, we also examined the impact of our results to different baseline conditions for hemoglobin concentration and its saturation.

## Methods

### Animal surgery for awake head-fixed imaging

Two strains of mice were used in these experiments: *B6.*129*P2-Pvalb* ^*tm1(cre)Arbr*^ */J*, and *Tg(Thy1-jRGECO1a)GP8.*31*Dkim/J*. Both strains were obtained from Jackson Laboratories (Bar Harbor, ME). The genetic background of these transgenic mice is not relevant to this study, but it forms an important aspect of our follow-up study. Procedures performed on the animals followed an experimental protocol approved by the University of Pittsburgh Institutional Animal Care and Use Committee (IACUC), and in accordance with the standards for humane animal care and use as set by the Animal Welfare Act and the National Institutes of Health Guide for the Care and Use of Laboratory Animals. All animals underwent an initial surgical procedure to place a cranial window for awake imaging several weeks following recovery. For cranial window surgery, animals were anesthetized using ketamine (75 mg kg^−1^) and xylazine (10 mg kg^−1^). They were then placed in a stereotaxic frame (Narishige, Tokyo, Japan) and supplementary oxygen in air (1:1) was administered at a rate of 500 ml min^−1^ using a nose cone (Narishige, Tokyo, Japan). Body temperature was maintained at 37 °C during the duration surgery using a heating blanket with temperature feedback (40–90- 8C; FHC, Inc., Bowdoinham, ME, USA). Surgery consisted of resecting the skin to expose the skull's parietal bone over the somatosensory cortex. Vetbond and dental cement were used to affix an aluminum head bar to the skull. Our head bar has an 8-mm diameter opening in the center which was placed over the parietal bone. A craniotomy was then performed using a dental drill over an area slightly larger than 4 mm in diameter, positioned about 2.5 mm lateral and 1.5 mm posterior from Bregma. A custom cover glass assembly consisting of a 4 mm round cover glass glued over a 5 mm round cover glass (CS-4R and CS-5R, Warner Instruments Inc.) was cemented onto the skull to seal the craniotomy while maintaining visual access of the brain. Following post-surgical care and recovery, animals were then returned to their cage and allowed to recover for 2–3 weeks following surgery. During the recovery period, mice were acclimated to our custom treadmill for awake head-fixed imaging by placing them in the treadmill for increasing time intervals each day, starting approximately 2 weeks after surgery.

### Optical imaging and laser doppler flowmetry

**Figure 1.**
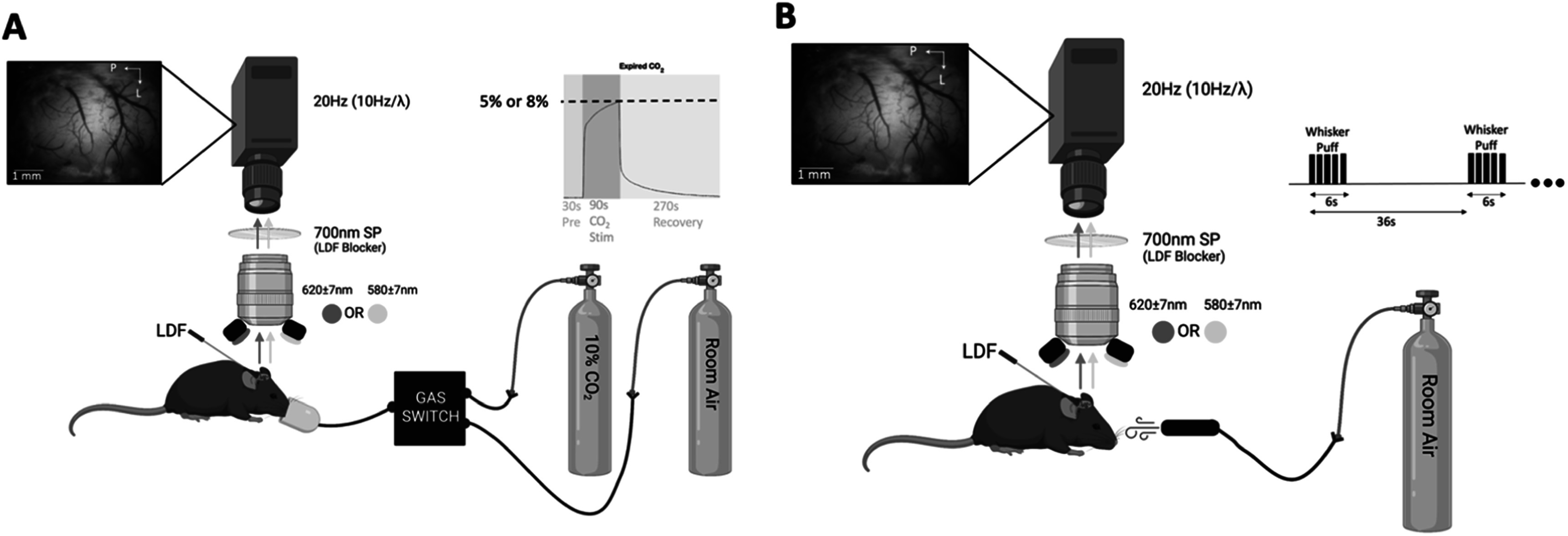
Experimental and imaging setup for the (A) CO_2_ delivery experiment, and (B) the whisker stimulation experiment. Generated with BioRender.com.

A custom optical imaging system was used to capture changes in the hemoglobin oxygenation state across the brain. Optical imaging of intrinsic signals (OIS) was conducted using a digital cooled-CCD camera (CoolSnap HQ2; Photometrics, Princeton, NJ, USA) through a macroscope (MVX-10; Olympus, Tokyo, Japan) at 2.5× magnification. Each frame was sequentially illuminated with filtered light at 580 ± 7 nm and 620 ± 7 nm using light emitting diodes, yielding an effective frame rate of 10 Hz per color. These wavelengths were chosen because of their relative sensitivity to total hemoglobin (HbT) and deoxygenated or reduced hemoglobin (HbR), respectively. A laser doppler flow probe (Periflux 5000/411, Perimed AB, Jarfalla, Sweden) was used to assess CBF concurrently with imaging. This unit operates at 785 nm and was placed facing sensory cortex at a 60-degree angle to minimize obstruction of the brain window (see figures 1(A), (B)). A short-pass optical filter (<700 nm) was placed along the imaging path to block LDF illumination from the camera. The LDF measurements were sampled at 100 Hz. For experiments where CO_2_ gas was delivered, we used a capnometer (Capnomac Ultima, Datex-Ohmeda Inc., Madison, WI) to measure end-tidal CO_2_ to ensure the desired gas concentration was delivered.

### Hypercapnia and whisker stimulation

A 10% CO_2_ enhanced air mixture was used to deliver hypercapnia stimulation at either 5% CO_2_ (7 animals) or 8% CO_2_ (10 animals). The stimulation paradigm consisted of 30 s of rest, 90 s of CO_2_ stimulation, and 270 s of recovery. During rest and recovery, the animal was administered medical-grade air. Imaging experiments were conducted for each CO_2_ level about 10 min apart. We also conducted whisker stimulation imaging experiments using air puffs to compare CMRO_2_ calculations of the evoked neuronal activity. Air puffs (50 ms in duration) were delivered using a pressure injector (Toohey Spritzer, Toohey Company, Fairfield, NJ) with a pressure of 40–50 psi. Stimulation for 6 s at frequencies of 5 and 10 Hz were repeated every 36 s, a total of 12 times per experiment in each animal. The data from the 12 trials from each stimulation frequency were averaged to extract average time series. Each stimulation paradigm (5 Hz and 10 Hz) was conducted in 7 animals. The complete experimental setup is shown in figure 1.

### Data analysis

All data were analyzed using MATLAB (MathWorks, MA, USA), and statistics were computed using Prism (GraphPad, CA, USA). In the main text we report values as mean ± standard deviation. The data from the LDF probe was downsampled to 10 Hz to match the imaging data. Camera images were then motion and intensity corrected. Motion correction consisted of a 2D Fourier-based sub-pixel, translation-only, rigid-body algorithm using the first image from each experiment as reference. Intensity correction was performed on all images by regressing out the average intensity from regions placed over the skull bone or head bar from each pixel in the image. This was done to remove unwanted fluctuations that might stem from the illumination system. Imaging and LDF data were concurrently recorded during both hypercapnia and whisker stimulation experiments. A region of interest was established for each animal for time series extraction using an oval (about 0.75 × 0.3 mm) over the LDF sampling area. For CO_2_ experiments, average signal changes were normalized by the average signal over the 30 s period prior to CO_2_ stimulation. The signal change consisted of the average normalized change over the 30 s period at the end of CO_2_ stimulation. For whisker stimulation experiments, all signals were normalized by the average 5 s period preceding whisker stimulation. The signal changes reported were obtained as the average 2 s change centered at the peak following stimulation onset. All data collected were used to compute the results presented in this paper, and no animals were excluded.

### CMRO_2_ calculation

To compute the relative change in CMRO_2_ (i.e. rCMRO_2_), we used Fick’s law under steady-state conditions, which can be written as rCMRO_2_ = rCBF · rOEF, where ‘r’ indicates the ratio relative to pre-stimulation baseline (rX = X/X_0_). Imaging data were then used to obtain the change in oxygen extraction fraction (OEF), where rOEF = (1−*C*_a_/*C*_v_)/(1−*C*_a,0_/*C*_v,0_) = (1−*S*)/(1−*S*_0_) and *S* is the blood oxygen saturation. To relate the imaging data to the changes in blood oxygen extraction fraction, the modified Beer–Lambert law (MBLL) is used to calculate the changes in the concentration of oxy-hemoglobin (∆*C*_HbO_) and deoxy-hemoglobin (∆*C*_HbR_) to changes in light absorption (∆*A*) at particular wavelengths (i.e. *λ*_1_, *λ*_2_) through their respective light extinction coefficients (*ε*’s) and light pathlength factors (*D*’s) (Dunn *et al* 2005, Ma *et al* 2016) (equation (1)). The changes in light absorption were obtained from the optical imaging data at a given wavelength as ${\mathrm{\Delta }}{A}_{\lambda }$ $=-\mathrm{ln}{\mathrm{}}(\frac{{I}_{\lambda }\left(t\right)}{{I}_{\lambda ,0}}),$ where *I* is the measured image intensity in the region of interest. The values for *ε* and *D* were obtained from (Ma *et al* 2016). Because most blood oxygen is bound to hemoglobin, the blood oxygen extraction fraction (OEF) is equal to *C*_HbR_/*C*_HbT_. The total oxygen concentration is the sum of oxy- and deoxygenated oxygen concentrations (*C*_HbT_ = *C*_HbO_ + *C*_HbR_). Considering that the oxygen concentration *C*_X_ = *C*_X,0_ + ∆*C*_X_, the expression to calculate rCMRO_2_ simplifies to equation (3) (Dunn *et al* 2005). We assumed a baseline concentration of total hemoglobin of 280 *μ*m (*C*_HbT,0_) and a baseline blood oxygen saturation of 75% (*S*_0_). As a result, optical measurements of ∆*C*_HbR_ and ∆*C*_HbT_ along with measurements of rCBF are sufficient to calculate rCMRO_2_ using equation (2). In this work, we aim to test whether equation (2) is sufficient to predict no changes in oxygen metabolism when using a known isometabolic stimulus (hypercapnia, rCMRO_2_ = 1) as well as testing the impact of assuming values for some of these parameters (described below) on rCMRO_2_ calculations (Kety and Schmidt 1948, Novack *et al* 1953, Krnjevic *et al* 1965, Nilsson and Siesjo 1976, Zappe *et al* 2008).

\begin{eqnarray*}\left[\begin{array}{l}{\unicode{x02206}A}_{\lambda 1}\\ {\unicode{x02206}A}_{\lambda 2}\end{array}\right]=\left[\begin{array}{cc}{{\mathrm{\epsilon }}}_{\mathrm{HbO},\lambda 1}{D}_{\lambda 1} &amp; {{\mathrm{\epsilon }}}_{\mathrm{HbR},\lambda 1}{D}_{\lambda 1}\\ {{\mathrm{\epsilon }}}_{\mathrm{HbO},\lambda 2}{D}_{\lambda 2} &amp; {{\mathrm{\epsilon }}}_{\mathrm{HbR},\lambda 2}{D}_{\lambda 2}\end{array}\right]\left[\begin{array}{l}{\unicode{x02206}C}_{\mathrm{HbO}}\\ {\unicode{x02206}C}_{\mathrm{HbR}}\end{array}\right]\end{eqnarray*}

\begin{eqnarray*}{\mathrm{rCMRO}}_{2}=\mathrm{rCBF}\displaystyle \frac{1+\tfrac{\unicode{x02206}{C}_{\mathrm{HbR}}}{{C}_{\mathrm{HbR},0}}}{1+\tfrac{\unicode{x02206}{C}_{\mathrm{HbT}}}{{C}_{\mathrm{HbT},0}}}.\end{eqnarray*}

As described, equation (2) is prone to error that stems from variations in partial volume of oxygenation signals. To account for spatial variations in vascular content, equation (2) is typically modified to include fractional coefficients that capture the volume fraction of blood occupied by deoxy-hemoglobin (*γ*_R_ = (∆*C*_HbR,venous_/∆*C*_HbR_)/(*C*_HbR,venous_/*C*_HbR_)) and total-hemoglobin (*γ*_T_ = (∆*C*_HbT,venous_/∆*C*_HbT_)/(*C*_HbT,venous_/*C*_HbT_)) (equation (3)) (Mayhew *et al* 2000). These parameters are typically assumed to take the value of 1 but can be difficult to measure.

\begin{eqnarray*}{\mathrm{rCMRO}}_{2}=\mathrm{rCBF}\displaystyle \frac{1+{\gamma }_{R}\tfrac{\unicode{x02206}{C}_{\mathrm{HbR}}}{{C}_{\mathrm{HbR},0}}}{1+{\gamma }_{T}\tfrac{\unicode{x02206}{C}_{\mathrm{HbT}}}{{C}_{\mathrm{HbT},0}}}(\mathrm{Model}1)\end{eqnarray*}

Often, imaging data is also used to estimate the changes in rCBF using measurements that relate changes in cerebral blood volume (rCBV) to rCBF using a power law (i.e. rCBV = rCBF^*α*^) (Grubb *et al* 1974). This equation is referred to as Grubb’s relationship and it simplifies equations (2)–(4) or equation (5), depending on whether rCBF measurements are available. The coefficient *k* (equation (5)) is usually called the calibration coefficient, and, for optical data, it intends to capture the baseline concentration of deoxy-hemoglobin as well as *γ*_R_ and other model assumptions. It is evident from equations (3)–(5) that a number of assumptions and measurements can be used to calculate rCMRO_2_, and these equations will be used to test three models below. Our goal is to test for differences among these models using Model 1 (equation (3)) with rCBF and optical data as our standard of reference.

\begin{eqnarray*}{\mathrm{rCMRO}}_{2}={\left(1+\displaystyle \frac{\unicode{x02206}{C}_{\mathrm{HbT}}}{{C}_{\mathrm{HbT},0}}\right)}^{1/\alpha }\cdot \,\displaystyle \frac{1+{\gamma }_{R}\tfrac{\unicode{x02206}{C}_{\mathrm{HbR}}}{{C}_{\mathrm{HbR},0}}}{1+{\gamma }_{T}\tfrac{\unicode{x02206}{C}_{\mathrm{HbT}}}{{C}_{\mathrm{HbT},0}}}\,\,(\mathrm{Model}2)\end{eqnarray*}

\begin{eqnarray*}{\mathrm{rCMRO}}_{2}={\mathrm{rCBF}}^{1-\alpha }\left(1+k\unicode{x02206}{C}_{\mathrm{HbR}}\right)\,\,(\mathrm{Model}3)\end{eqnarray*}

We describe different input data scenarios consisting of the number of wavelengths used during imaging (i.e. 2 or 1) and either inclusion or estimation of rCBF measurements. When not supplied we estimated rCBF using rCBF = rCBV^1/*α*^ = rC_HbT_^1/*α*^. Calibrated and uncalibrated approaches were tested, where a calibrated approach involved using hypercapnia data from each animal to compute rCMRO_2_, and uncalibrated approaches involved using either the average value from the calibrated model or a fixed number from literature. Models that are provided 2 imaging wavelengths (WL) enable the computation of *C*_HbO_ and *C*_HbR_, while models that are given 1 WL, we supply only 620 nm data since it is mostly sensitive to ∆*C*_HbR_. In more detail, these were the models tested.

#### Model 1 (2WL+LDF)

We consider this to be our most complete model (equation (3)). We evaluate three cases. In the first case (Model 1-Cal), we estimate the parameters *γ*_R_ and *γ*_T_ for each animal using the optical and LDF data from hypercapnia stimulation to ensure rCMRO_2_ = 1. Because the solution to this parameter set is not unique, we select the paired solution that is closest to (1, 1) in (*γ*_R_ , *γ*_T_) space. We will use this case as our standard of reference across cases and models. In the second case (Model 1-Avg), we test the impact of using the average *γ*_R_ and *γ*_T_ values across animals on our calculated rCMRO_2_ values. For the third case (Model 1–1), we evaluate the impact of assuming *γ*_R_ and *γ*_T_ = 1 on rCMRO_2_. We consider the last two cases as uncalibrated since we used fixed values for *γ*_R_ and *γ*_T_.

#### Model 2 (2WL-only)

For this model we test the same three cases described for Model 1. We refer to these models as Model 2-Cal, Model 2-Avg and Model 2–1. Since LDF measurements are not included in this model, rCBF is estimated using rCHbT from the imaging data. The exponent *α* was computed for each animal using the rCBF and rCHbT to compare it against literature values (Grubb *et al* 1974). Here we also consider the last two cases (Model 2-Avg and Model 2–1) as uncalibrated.

#### Model 3 (1WL+LDF)

This model is motivated by the magnetic resonance imaging (MRI) method of computing rCMRO_2_ (Davis *et al* 1998, Hoge *et al* 1999b), where arterial spin labeling (ASL) and blood-oxygenation level dependent (BOLD) MRI signals are used as measurements of changes in blood flow and *C*_HbR_, respectively. In this model, we use rCBF measurements to estimate r*C*_HbT_ and assume that 620 nm imaging is a close approximation of r*C*_HbR_ (i.e. r*C*_HbR_ ≈ 1+∆*A*_620_). Similar to calibrated fMRI, calibration is used to calculate the *k* in equation (5) from hypercapnia data (Model 3-Cal). Similar to Models 1 and 2, we compare three cases relative to Model 3-Cal, one using the average calibration coefficient across animals (Model 3-Avg), and another case where the actual r*C*_HbR_ is used instead of that approximated by 620 nm imaging data (Model 3-Ideal). Here, only Model 3-Avg and Model 3-Ideal are uncalibrated, since it uses the average calibration values across all animals.

To test for statistically significant differences between the computed rCMRO_2_ of the different models, we report the results of paired t-tests compared to our rCMRO_2_ reference (Model 1-Cal).

## Results

### CO_2_ Calibration

**Figure 2.**
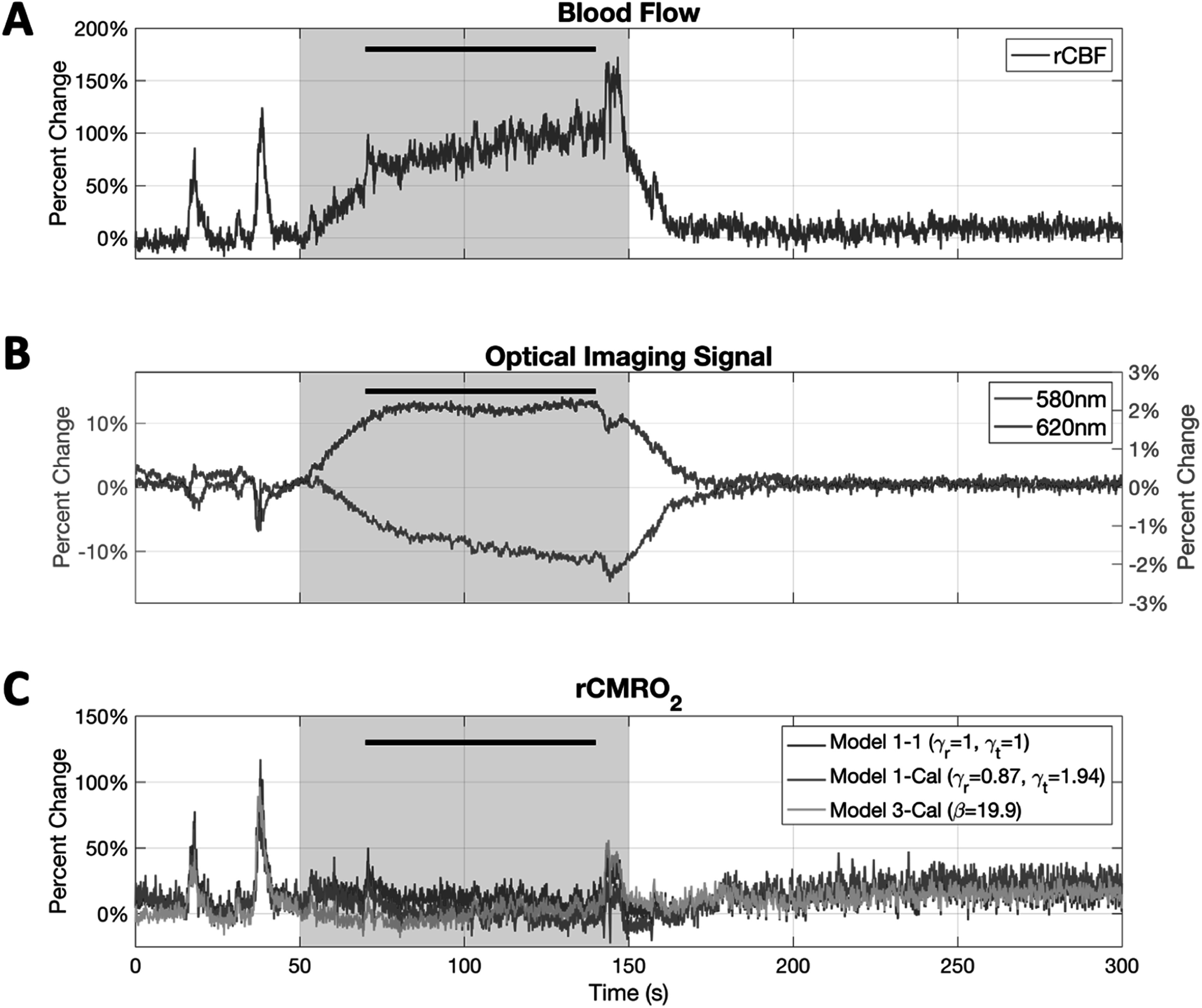
Cerebral blood flow, OIS-580 nm, OIS-620 nm and computed rCMRO_2_ in one representative animal after 8% CO_2_ administration. Shading represents the period of CO_2_ delivery, and the bar represents the time period in which the mean was taken over to compute the calibration coefficients. (A) Measured rCBF signal from LDF probe. (B) Mean 580 nm and 620 nm signal in the LDF probe region. The left axis denotes the 580 nm signal change, and the right axis denotes the 620 nm signal change. (C) rCMRO_2_ computed using the Model 1–1 (2WL+LDF) and Model 1-Cal (2WL+LDF) and Model 3-Cal (1WL+LDF). As expected, both calibrated models show no changes in CMRO_2_ during hypercapnia, while the uncalibrated Model 1–1 has a positive offset. The transient changes observed in the LDF and OIS data stem from the awake head-fixed nature of the experiments where the animals were not sedated during these experiments and these signals increased or decreased.

Experiments were successfully conducted in all animals under awake head-fixed conditions after acclimation to our imaging setup. Animals were administered two concentrations of CO_2_, 5% and 8%, to test the effect of different isometabolic CBF changes on Models 1, 2 and 3. Hypercapnia gas administration induced global signal changes over the imaging window; here we report values from the LDF probe ROI. Figure 2 shows the change in optical imaging signals, rCBF and rCMRO_2_ from a representative animal after 8% CO_2_ administration.

During 5% CO_2_, the average signal change in OIS-580 nm and OIS-620 nm signal were −4.28 ± 1.67% and 1.27 ± 0.24%, respectively, yielding computed changes in HbR of −16.60 ± 3.71% and HbO of 20.59 ± 6.27%. The CBF change measured by LDF was 29.17 ± 10.62%. During 8% CO_2_ delivery, the OIS-580 nm signal changed by −13.40 ± 4.12% and the OIS-620 nm signal changed by 2.01 ± 0.77%, yielding changes in HbR and HbO of −37.48 ± 8.91% and 59.37 ± 17.8%, respectively. CBF increased by 113.67 ± 39.44%. We used these values to compute values for *γ*_R_ and *γ*_T_ for each animal to ensure rCMRO_2_ = 1 for Model 1-Cal (2WL+LDF) and Model 2-Cal (2WL). For 5% CO_2_ administration, we obtained *γ*_R_ and *γ*_T_ values of 0.73 ± 0.07 and 1.35 ± 0.18, respectively. CO_2_ calibration of Model 2-Cal (2WL) computed the same *γ*_R_ and *γ*_T_ values as Model 1-Cal (2WL+LDF). For Model 3-Cal (1WL + LDF), the calibration factor *k* was 11.23 ± 2.42. These data predicted a Grubb’s exponent (*α*) of 0.38 ± 0.06. For the 8% CO_2_ stimulation data, we obtained Model 1-Cal (2WL+LDF) and Model 2-Cal (2WL-only) values for *γ*_R_ and *γ*_T_ of 0.72 ± 0.08 and 1.64 ± 0.31, respectively. For Model 3-Cal (1WL + LDF), we obtained an average value of 16.47 ± 3.83 for the calibration factor k. The mean value for the Grubb exponent for these data was 0.38 ± 0.06.

Since the calibration coefficients as well as Grubb’s exponent should be similar over regions that samples numerous arteries, capillaries and veins, we averaged the coefficients from 5% and 8% CO_2_ delivery across mice average *γ*_R_ and *γ*_T_ values of 0.72 (± 0.08) and 1.52 (± 0.29), respectively. These values were then used for Model 1-Avg (2WL+LDF) and Model 2-Avg (2WL-only). For Model 3-Cal (1WL+LDF), the average calibration factor *k* value was 14.32 (± 4.18). The average Grubb exponent *α* was 0.38 (± 0.06).

**Figure 3.**
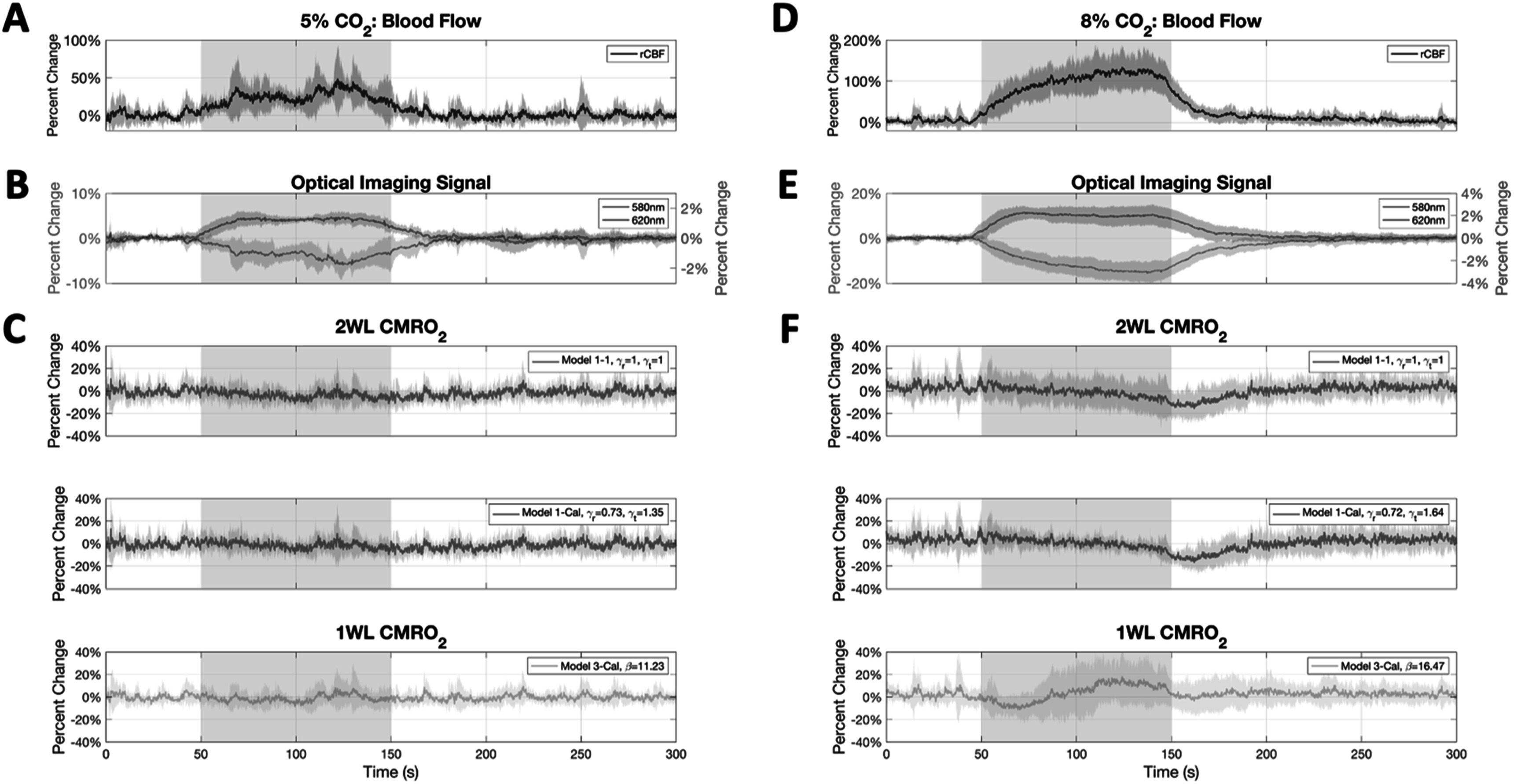
Average time series of measured optical signals and computed rCMRO_2_ during CO_2_ administration. Each time series shows the mean of all animals in the experiment (*n* = 7 for 5% CO_2_, *n* = 10 for 8% CO_2_), and shading denotes standard deviation. The gray shaded box denotes the time during which CO_2_ was delivered. (A) Change in cerebral blood flow (CBF) during 5% CO_2_ delivery, (B) change in OIS-580 nm (left axis) and OIS-620 nm signal (right axis) during 5% CO_2_ delivery, and (C) change in computed rCMRO_2_ during 5% CO_2_ delivery using the uncalibrated Model 1–1 (2WL+LDF), calibrated Model 1-Cal (2WL+LDF), and calibrated Model 3-Cal (1WL+LDF). (D)–(F) Show the corresponding changes in CBF, OIS-580 nm, OIS-620 nm and rCMRO_2_ during 8% CO_2_ delivery. Although all cases show no significant changes in rCMRO2 to hypercapnia stimulation, the 5% CO_2_ data shows less variability during the stimulation period.

By design, Model 1-Cal (2WL+LDF), Model 2-Cal (2WL-only) and Model 3-Cal (1WL+LDF) met our condition of rCMRO_2_ = 1 over the selected 30 s period during CO_2_ stimulation. Fixing gamma values to 1 during CO_2_ administration (uncalibrated models) yielded deviations in rCMRO_2_ of −2.99 ± 8.02% for Model 1–1 (2WL+LDF) and −2.36 ± 5.12% for Model 2–1 (2WL-only). None of these rCMRO_2_ values were significantly different from 0 using a two-sided t-test (*p* > 0.08). Using the average *γ*_R_ and *γ*_T_ yielded differences in rCMRO_2_ values of −0.25 ± 7.41% and 0.44 ± 4.49% for Model 1-Avg (2WL+LDF) and Model 2-Avg (2WL-only), respectively. Neither of these values were significantly different from 0 (two-sided t-test with *p* > 0.69). Similarly, the difference in rCMRO_2_ computed by Model 3-Avg (1WL+LDF) was 1.80 ± 13.58% (not significantly different from 0, *p* > 0.59). The average CBF, optical imaging signals and rCMRO_2_ traces from hypercapnia stimulation at 5% and 8% CO_2_ are shown in figure 3.

### Whisker stimulation

**Figure 4.**
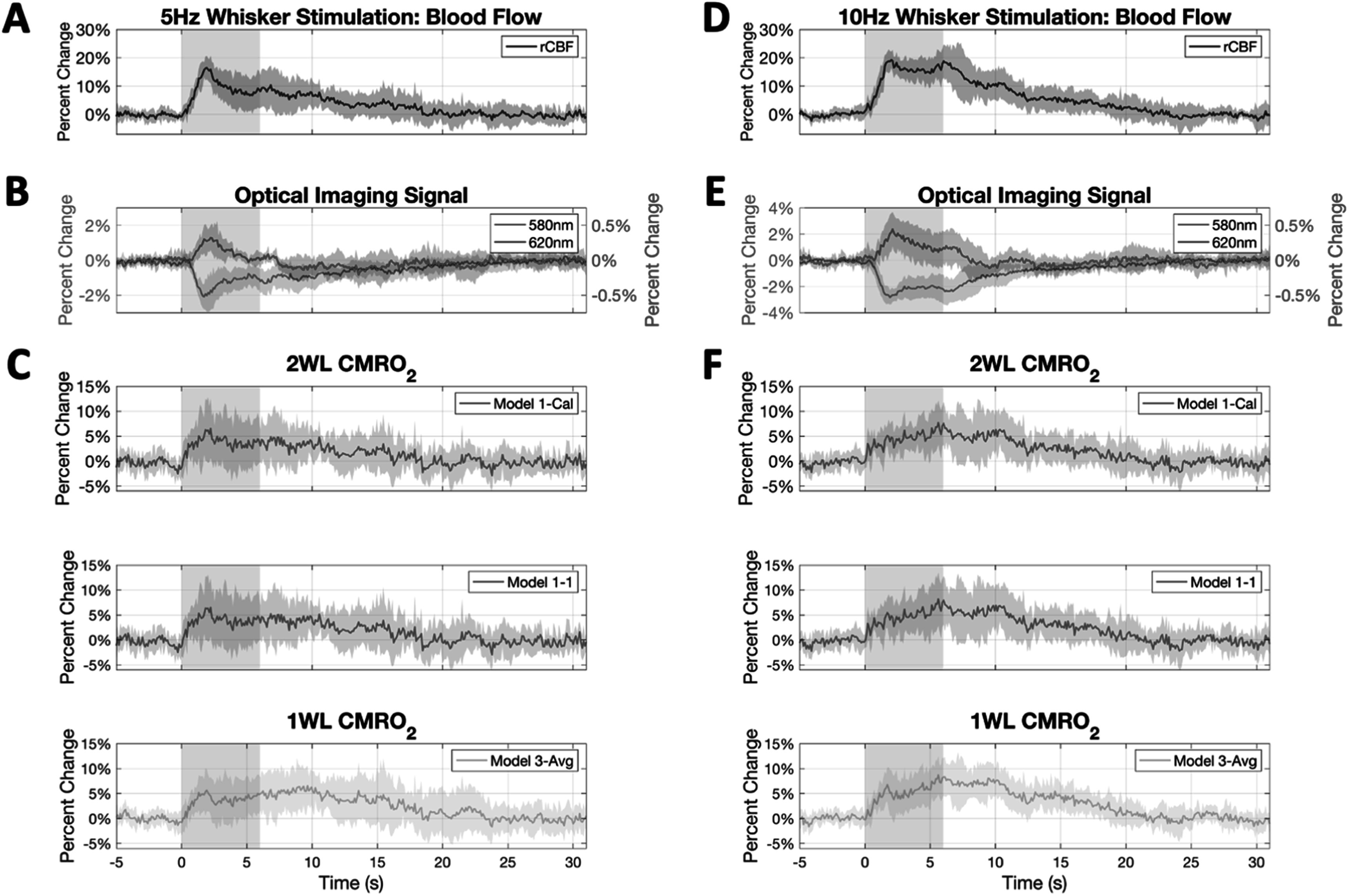
Average time series of measured optical signals and computed rCMRO_2_ during whisker stimulation (*n* = 7 for both 5 Hz and 10 Hz whisker stimulation). The colored shading denotes standard deviation. The gray shaded box denotes the time during stimulus delivery. (A) Change in cerebral blood flow (CBF) to 5 Hz stimulation along with the (B) change in OIS-580 nm (left axis) and OIS-620 nm signals (right axis), as well as the (C) computed rCMRO_2_ change to 5 Hz stimulation using calibrated Model 1-Cal (2WL+LDF), uncalibrated Model 1–1 (2WL+LDF), and calibrated Model 3-Cal (1WL + LDF). (D)–(F) Show the corresponding changes in rCBF, OIS-580 nm, OIS-620 nm and rCMRO_2_ to whisker stimulation at 10 Hz.

Whisker stimulation at 5 Hz produced average OIS-580 nm and OIS-620 nm signal changes of −1.11% ± 0.48% and 0.14% ± 0.11%, respectively, and 10 Hz whisker stimulation produced changes of −1.57% ± 0.65% and 0.17% ± 0.17%, respectively, in our mice (*n* = 7). The average CBF increase for 5 and 10 Hz whisker stimulation were 13.79 ± 4.07% and 17.40 ± 3.45%, respectively. We then used our CO_2_ calibration results to compute rCMRO_2_ changes produced by whisker stimulation using the different model scenarios and compared these rCMRO_2_ computations to our reference model (Model 1-Cal). The average rCMRO_2_ computed as a result of 5 and 10 Hz whisker stimulation were 5.16 ± 5.63% and 5.91 ± 2.32%, respectively, and both significantly larger than 0 (*p* < 0.026). Figure 4 shows the mean time traces across animals for rCBF and rCMRO_2_.

We then compared rCMRO_2_ results with calibration (Model 1-Cal) to uncalibrated Model 1-Avg and Model 1–1. We observed lower error for Model 1-Avg (2WL+LDF) compared to Model 1–1 (2WL+LDF) (7% versus 9%), suggesting that using average *γ* values slightly contribute to correcting rCMRO_2_. We also compared calibrated rCMRO_2_ values obtained without rCBF measurements from Model 2-Cal (2WL-only) to uncalibrated Model 2-Avg and Model 2-1. We found that although the errors between Model 2-Cal and Model 2-Avg were similar to those above (11.5% on average), the computed rCMRO_2_ significantly differed from that of Model 1 cases by a larger margin (error between 61% and 72%). Paired t-tests were conducted for the results produced from each model across mice. Significant differences in rCMRO_2_ were found between Model 1-Cal and Model 2-Cal (*p* = 0.010), Model 2-Avg (*p* = 0.003) and Model 2–1 (*p* = 0.004).

Next, we compared the percent error between Model 1-Cal (2WL+LDF) and Model 3 (1WL+LDF) rCMRO_2_ calculations. Overall the error was lower compared to Model 2, with Model 3-Cal and Model 3-Ideal showing lower error levels. No significant differences were observed between Model 1-Cal (2WL+LDF) and Model 3-Cal (1WL+LDF) and Model 3-Avg (1WL+LDF) except for Model 3-Ideal (1WL+LDF; *p* = 0.020), even though that one showed the lowest error.

**Figure 5.**
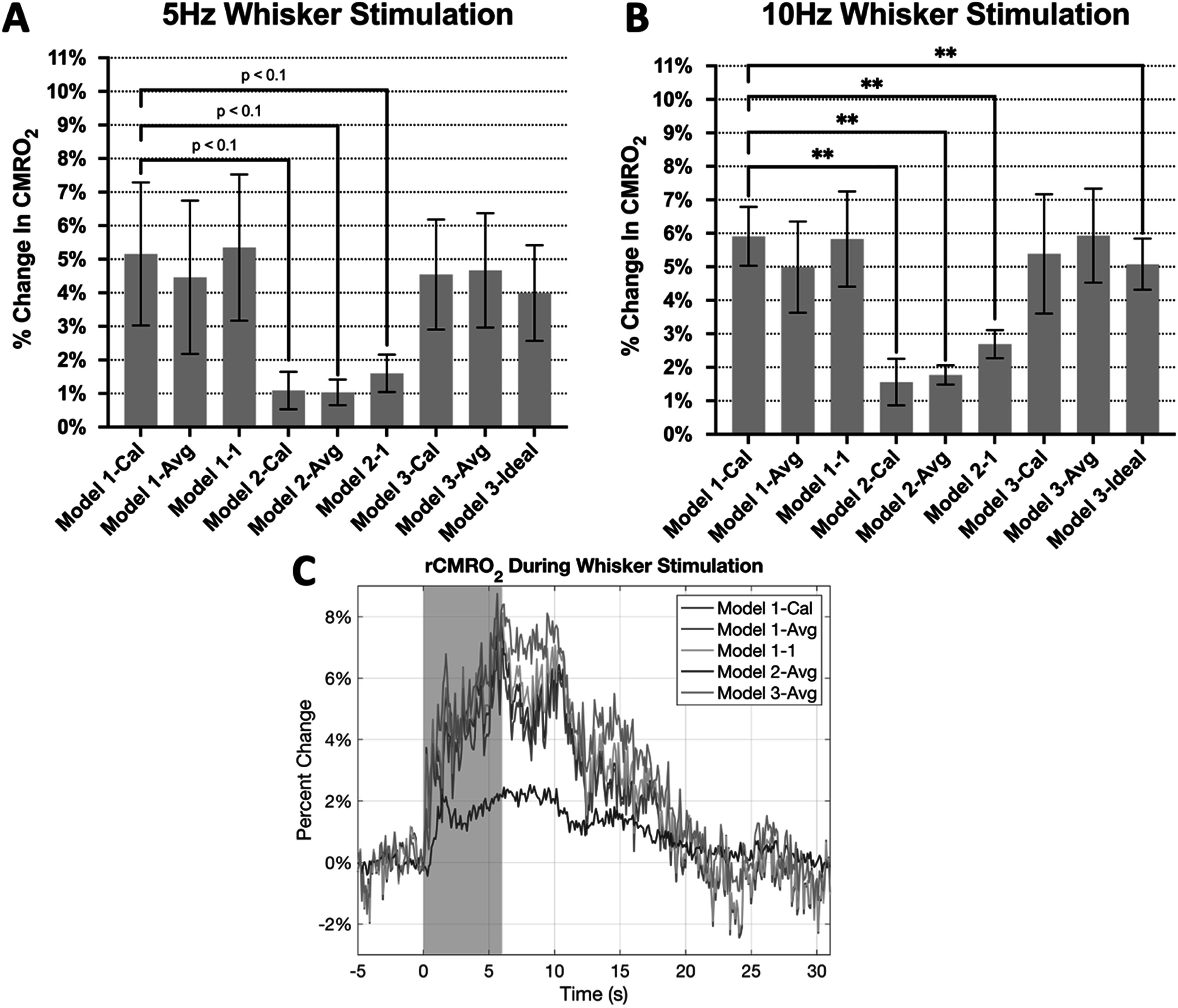
Comparison of rCMRO_2_ values calculated from the different models (Model 1 (2WL+LDF), Model 2 (2WL-only), Model 3 (1WL+LDF)). (A) Changes in rCMRO_2_ to 5 Hz whisker stimulation (error bars denote standard error). (B) Change in rCMRO_2_ to 10 Hz whisker stimulation. Statistical significance was established using paired t-tests (*p* < 0.05). (C) Average r**C**MRO_2_ time series to 10 Hz whisker stimulation for several different models. Small differences are observed between the models except for Model 2, indicating that CBF measurements are important for rCMRO_2_ computation.

The change in CMRO_2_ for the whisker data was extracted as the 2 s time series average around its peak and shown in figures 5(A) and (B). We observe the largest deviations between the models to take place between Model 1 and Model 2, where Grubb’s expression is used as the estimate for blood flow. Figure 5(C) shows time series of several model computation methods using the mean 10 Hz whisker stimulation time series. As seen in this figure, there appears to be minimal temporal difference between the different cases.

### Effect of the baseline hemoglobin concentrations

**Figure 6.**
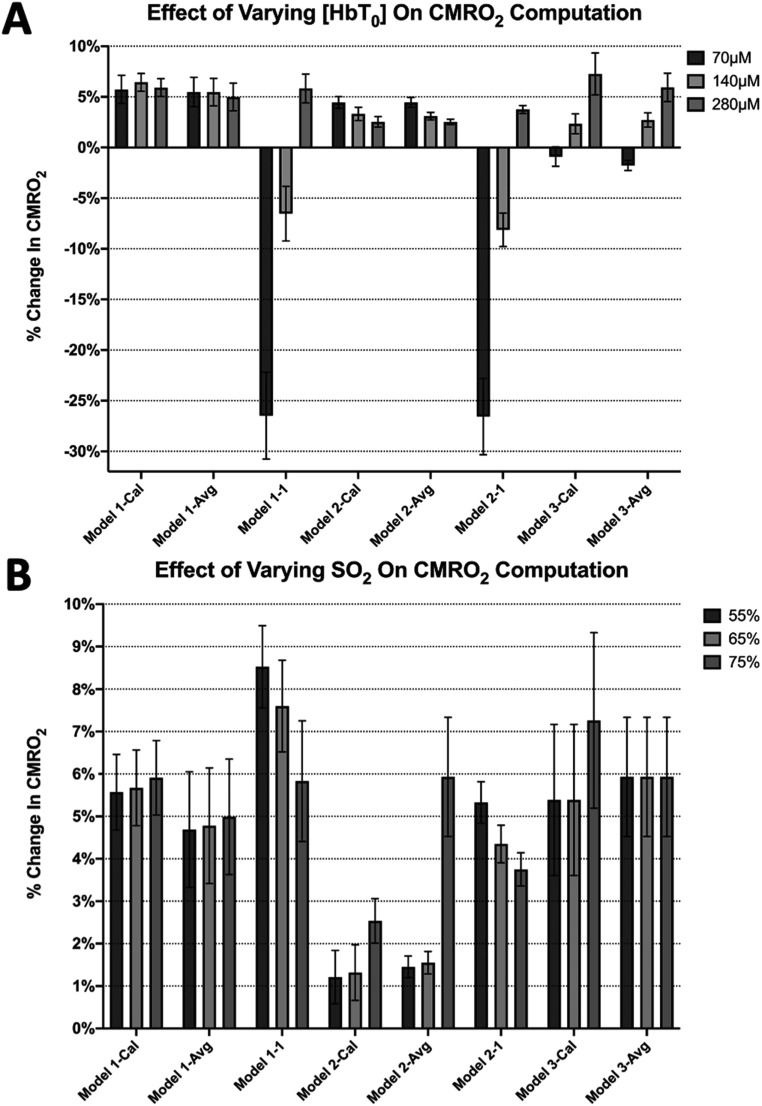
Impact of the initial concentration of total hemoglobin (HbT) on rCMRO_2_ calculations. (A) The mean rCMRO_2_ values using the four models listed . An oxygen saturation of 75% was assumed. Error bars represent the standard error. (B) The mean rCMRO_2_ values using the four models listed at different *S*_O2_ values. An initial HbT concentration of 280 *μ*M was used. It is evident that *C*_HbT,0_ has a great impact on the percent error between rCMRO_2_ computation methods, while varying oxygen saturation (*S*_O2_) has much less of an effect.

To determine how our results are influenced by the baseline concentration of hemoglobin and oxygen saturation, we computed rCMRO_2_ for the 10 Hz whisker stimulation data using *C*_HbT, 0_ of 70 *μ*M, 140 *μ*M and 280 *μ*M (all assuming 75% oxygen saturation). We also used these data to compute rCMRO_2_ with initial oxygen saturation of 75%, 65% and 55% (all *C*_HbT, 0_ of 280 *μ*M). The summary of these results is shown in figures 6(A) and (B).

The calibration factors were recomputed for several different initial (i.e. baseline) hemoglobin concentrations to compute changes in CMRO_2_. Upon inspection of these results, changing the baseline hemoglobin concentration produced larger differences in rCMRO_2_ than adjusting the baseline oxygen saturation across model scenarios, while the range of rCMRO_2_ changes was least impacted by Model 1-Cal (2WL+LDF). We observe that different initial hemoglobin concentrations led to larger differences in rCMRO_2_ in Models 1–1 and 2-1 (uncalibrated), and less drastic changes for Model 3-Cal and 3-Avg (1WL+LDF). Interestingly, using Model 1-Cal (2WL+LDF) and Model 1-Avg (2WL+LDF) yielded similar results, regardless of initial hemoglobin concentration or initial oxygen saturation. Model 1–1 and Model 2 showed the largest effect when changing the initial oxygen saturation. As expected, since Model 3 does not require using the modified Beer–Lambert’s law, it showed no effect on rCMRO_2_ computation when varying the initial oxygen saturation.

## Discussion

In this study we evaluated the impact of calibrated and uncalibrated models to compute relative changes in the cerebral metabolic rate of oxygen metabolism (rCMRO_2_) from hemoglobin-sensitive optical imaging in awake head-fixed mice. We tested three rCMRO_2_ model scenarios using two different types of stimulation data. Our experiments show that these models are relatively robust when using hypercapnia stimulation data since they produced nearly no significant changes in rCMRO_2_ for the uncalibrated models we tested, although some bias to underpredict rCMRO_2_ was observed (less than 3%). Calibration ensured no changes in rCMRO_2_ were obtained during CO_2_ administration and average values for *γ*_R_ and *γ*_T_ of 0.72 and 1.52, respectively, were obtained for Models 1 (2WL+LDF) and 2 (2WL-only). For Model 3 (1WL+LDF), the average calibration factor *k* was 14.32. Interestingly, the average value for Grubb’s coefficient in our study was 0.38. Whisker stimulation experiments at 5 and 10 Hz were then conducted to elicit sensory cortex activity and CBF increased by 13% to 17%, respectively. Calibrated Model 1 (Model 1-Cal) yielded a change in rCMRO_2_ of 5.5% and its uncalibrated forms (Model 1-Avg and Model 1–1) produced similar changes (within 9%). However, we did find a statistically significant underestimation of rCMRO_2_ when replacing the blood flow term with Grubb’s estimates (Model 2). Model 3, yielded results closer to Model 1 but with higher variability. We then tested the impact of initial conditions, and we observed more variability on Models 2 and 3 than Model 1, whether calibrated or not. Of these, the initial hemoglobin concentration had a stronger impact on rCMRO_2_ calculation than the baseline oxygen saturation; however, the calibrated forms of Model 1 and 2 were relatively insensitive to assumptions of the baseline condition.

### Hypercapnia as an isometabolic stimulus

Commonly used approaches to quantify CMRO_2_ in humans and animals rely on a biophysical model that is given non-invasive measurements of blood flow and blood oxygenation taken under two conditions (e.g. baseline versus stimulation). Because the biophysical model makes a number of assumptions, a calibration step is usually used to assign values to parameters instead of assuming them. This step requires known CMRO_2_ changes for model calibration. Hypercapnic stimulation has emerged as the preferred manipulation since it is non-invasive, produces blood flow changes similar to those produced by neuronal activity and has been previously tested to not alter oxidative metabolism or neuronal activity (Davis *et al* 1998, Hoge *et al* 1999a, 1999b, Goodwin *et al* 2009, Lajoie *et al* 2017). That said, other strategies including pharmacological administration of transient dilatory agents that do not alter neuronal activity could be used for calibration. Multiple studies have examined hypercapnia as isometabolic and have reported no significant change in neuronal metabolism (rCMRO_2_ = 1) during low grade (5%–7%) CO_2_ delivery (Kety and Schmidt 1948, Novack *et al* 1953, Krnjevic *et al* 1965, Nilsson and Siesjo 1976, Zappe *et al* 2008). In this study, we delivered 5% and 8% CO_2_ to the animals for calibration in awake animals. Previous studies, however, also report that when CO_2_ reaches 10%–12%, neuronal activity and metabolism are suppressed (Krnjevic *et al* 1965). Thus, we ensured that less than 10% CO_2_ was administered using the end-tidal reading from the capnometer. We found that 5% CO_2_ elicited increases in CBF of about 30% which is similar to neuronal evoked increases in CBF from sensory stimulation. Although increasing the CO_2_ concentration to 8% increased the CBF response, it did not really change the values we obtained for gamma or for the Grubb exponent, suggesting that calibration at 8% CO_2_ behaves similar to 5% CO_2_.

### Calibration

Our various comparisons of rCMRO_2_ calculations relied on Model 1-Cal (2WL+LDF) as our reference. We chose this model scenario because it contained the most measurements and the least number of assumptions or unknowns. Our results show that there is not that much difference on rCMRO_2_ calculation (less than 9%) between Model 1-Cal (2WL+LDF) and Model 1-Avg (2WL+LDF) or Model 1–1 (2WL+LDF). Not surprisingly, Model 1–1 (2WL+LDF) had the largest error, suggesting that using average values of *γ*_R_ and *γ*_T_ provided slight improvements. Our results also show that measurements of rCBF are very important when calculating rCMRO_2_ (and perhaps more important than calibration) since Model 2 performed worse than Model 3. Another important observation is that Model 1-Avg (2WL+LDF) was less sensitive to initial concentrations than Model 1–1 (2WL+LDF), suggesting that using average values *γ*_R_ and *γ*_T_ for can help reduce variability. Since we observed some bias to underestimate rCMRO_2_ based on the hypercapnia data, we expected a general underestimation of rCMRO_2_ for the whisker stimulation data when using uncalibrated models; however, this was not consistently observed. Perhaps the biggest advantage of calibration was reducing the variability in rCMRO_2_ calculations across animals (Model 1-Cal rCMRO_2_ values were the least variable). Since rCMRO_2_ involves combination of several measurements, the inherent noise or variability of each measurement only increases variability in the calculation, and steps to reduce this variability (like calibration) should be considered.

Although fMRI computations of rCMRO_2_ are generally calibrated, our results suggest that optical methods for measuring rCMRO_2_ may not need calibration. Our results suggest that if calibration is not possible or available, Model 1-Avg (2WL+LDF) does provide the closest rCMRO_2_ values to Model 1-Cal (2WL+LDF). Our average values for *γ*_R_ and *γ*_T_ of 0.72 and 1.52 are not a unique solution for these parameters from calibration data, we chose them as the solution that is closest to (1, 1). Another possibility is to fix one of these parameters to 1 (e.g. *γ*_R_ = 1) and solve for the other parameter (*γ*_T_). Because these parameters can have a spatial dependence (e.g. at the pixel level), spatial calibration may be desired if spatial calculations of CMRO_2_ are desired. Lastly, whether these different solutions have a different impact on CMRO_2_ remains to be evaluated.

### Evaluation of Grubb’s exponent

Grubb’s power law relates cerebral blood flow to cerebral blood volume (CBV). Our Grubb exponent calculations can be evaluated using arterial diameter measurements from the images to estimate the blood flow changes in cortex since these are dominated by changes in arterial diameter. Using wide-field imaging, we measured pial vessel diameters over the imaging window during 8% CO_2_ delivery and found a power relation between CBF and arterial diameter of 0.27 ± 0.10. This compares fairly well to Grubb *et al*’s findings of a power of 0.38 (Grubb *et al* 1974) since here we only considered arteries which would constitute a lower bound for this exponent from our data. Nonetheless, our computed value of 0.27 is close to Dunn *et al*’s findings, where they reported an exponent of 0.25 ± 0.03 during forepaw stimulation (Dunn *et al* 2005). In general, the range of this exponent has been reported to range between 0.18 and 0.38 (Mandeville *et al* 1999, Jones *et al* 2001, 2002, Sheth *et al* 2004). We examined the effects of using the average Grubb coefficient on the computation of rCMRO_2_, which consistently yielded significant differences from our reference model. This strongly suggests that direct measurements of rCBF significantly help reduce bias in rCMRO_2_ estimates.

### Effect of baseline conditions on rCMRO_2_

Baseline conditions have an effect on the calculated rCMRO_2_ as previously reported (Mayhew *et al* 2000, Dunn *et al* 2005). Additionally, optical properties used to compute CMRO_2_ such as the differential pathlength factor may differ depending on the experimental conditions (Lin *et al* 2015). Our examination of baseline conditions showed that the impact on CMRO_2_ was dependent on the amount of data given to the model, with calibrated models showing the least dependence on baseline concentrations. These results advocate for adding calibration data rCMRO2 experiments, especially for those where the baseline conditions might deviate from normal physiological conditions. In terms of its impact on our changes in CMRO_2_ values, our results are similar to those obtained from other groups using similar methods and different baseline concentrations. For example, Lee *et al* reported peak CBF and rCMRO_2_ changes of 7.94% and 3.46%, respectively, to optogenetic activation of pyramidal neurons, using 100 *μ*M as the initial concentration of HbT and 60% initial oxygen saturation (Lee *et al* 2021). Dunn *et al* reported a peak rCBF of 6% and peak rCMRO_2_ of 3% following whisker stimulation in lightly anesthetized rats with the same baseline conditions (Dunn *et al* 2005). Dahlqvist *et al* reported a rCBF change of 9% and rCMRO_2_ change of 4% to whisker pad stimulation in lightly sedated mice. Interestingly, they used a tissue pO_2_ probe which provides quantitative baseline information and essentially avoids strong dependence on baseline concentrations of hemoglobin (Dahlqvist *et al* 2020). Our calculated rCMRO_2_ changes to whisker stimulation in awake head-fixed mice produced slightly larger changes (about 5.5% on average) and also slightly larger CBF changes (13%–17%). These physiological changes are likely close to ‘true’ values since calibration helped ground the model to changes in blood oxygenation, and the aforementioned calibrated rCMRO_2_ values were fairly insensitive to baseline conditions.

In summary, our experiments show that the uncalibrated models we tested can be relatively robust since they produced essentially no changes in rCMRO_2_ when given hypercapnia isometabolic data. Calibration ensured no changes in rCMRO_2_ were obtained during CO_2_ stimulation, while whisker stimulation produced a change in rCMRO_2_ of 5.9% for our reference model (Model 1-Cal) and values of 5.0% and 5.8% for its uncalibrated forms (Model 1-Avg and Model 1–1, respectively). However, we did find a significant underestimation of rCMRO_2_ (between 1% and 3%) when replacing the blood flow term with Grubb’s estimates (Model 2-Cal, Model 2-Avg and Model 2–1) even though the Grubb exponent on average was similar to that previously reported. Including CBF measurements but using only one wavelength for optical imaging produced rCMRO_2_ changes between 4% and 6% but with larger standard deviation. We also tested the impact of initial conditions, and we observed more variability for Models 2 and 3 than Model 1, whether calibrated or not. Therefore, our work would recommend Model 1-Avg, Model 1–1 or Model 3-Cal as alternatives to Model 1-Cal.

### Limitations

The biggest limitation of our work is the lack of a direct measurement of CMRO_2_ in our experiments as a true reference. Direct measurements of CMRO_2_ are very difficult to conduct, especially in awake subjects. When considering non-invasive (or minimally invasive) approaches, the computation of CMRO_2_ relies on several assumptions about the underlying cerebral physiology, which likely introduce error into the computation of CMRO_2_. We note that our results (table 1, table 2) have large variability (high standard deviation), which can be attributed to the compounding error introduced by combining measurements in thecalculation of CMRO_2_. This high error was not driven by one particular animal and our conclusions are based on models that show the least variability. The first assumption is that all oxygen is carried by hemoglobin. This assumption, though is fairly reasonable, as 96%–98% of all oxygen is bound to hemoglobin under normal physiological conditions (Collins *et al* 2015). Another assumption is that arterial blood supplying our region of interest is fully oxygenated (arterial oxygen saturation is 100%). This assumption is reasonable at the level of pial vasculature. Another model assumption is that oxygen delivery and consumption are in continuous equilibrium (steady state). This implies that for every observation oxygen delivery is immediately consumed in tissue. Previous measurements suggest that equilibrium takes a few seconds (Vazquez *et al* 2012), such that this assumption does not present a major shortcoming. We tested the impact of initial conditions, since those likely have a larger impact that the assumptions discussed above. Notwithstanding, all these possible sources of error need to be considered when designing experiments that aim to calculate changes in CMRO_2_ from optical (or MRI) data.

**Table 1.** Percent error (mean ± standard deviation) in rCMRO_2_ to whisker stimulation between Models 1 and 2. The last two rows indicate the percent error in rCMRO_2_ models using the average data across animals.

Percent error	Model 1- Cal	Model 1-Avg	Model 1–1	Model 2-Cal	Model 2-Avg	Model 2–1
Across animals	Reference	6.75 ± 11.47%	9.13 ± 7.38%	71.09 ± 30.14%	72.16 ± 28.99%	61.32 ± 38.39%
Mean of 5 Hz	Reference	9.38%	1.28%	59.87%	60.86%	42.25%
Mean of 10 Hz	Reference	11.15%	7.12%	64.42%	64.68%	46.89%

**Table 2.** Percent error in rCMRO_2_ to whisker stimulation using Model 3 (1WL+LDF) and Model 1-Cal (2WL+LDF) as reference. The last two rows indicate the percent error in CMRO_2_ models using the average data across animals.

Percent Error	Model 1-Cal	Model 3-Cal	Model 3-Avg	Model 3-Ideal
Across animals	Reference	23.66 ± 20.86%	35.87 ± 49.06%	21.94 ± 10.74%
Mean of 5 Hz	Reference	6.32%	16.61%	21.52%
Mean of 10 Hz	Reference	12.18%	20.87%	8.86%

## Data Availability

The data that support the findings of this study are openly available at the following URL/DOI: https://github.com/neuroimlabpitt/CO2-Calibration.git.

## References

[pmeaad3a2dbib1] Acharya D (2022). Non-invasive spectroscopy for measuring cerebral tissue oxygenation and metabolism as a function of cerebral perfusion pressure. Metabolites.

[pmeaad3a2dbib2] Baker W B (2019). Continuous non-invasive optical monitoring of cerebral blood flow and oxidative metabolism after acute brain injury. J. Cereb. Blood Flow Metab..

[pmeaad3a2dbib3] Barrett M J, Suresh V (2015). Improving estimates of the cerebral metabolic rate of oxygen from optical imaging data. Neuroimage.

[pmeaad3a2dbib4] Berwick J (2005). Neurovascular coupling investigated with two-dimensional optical imaging spectroscopy in rat whisker barrel cortex. Eur. J. Neurosci..

[pmeaad3a2dbib5] Chong S H (2022). Real-time tracking of brain oxygen gradients and blood flow during functional activation. Neurophotonics.

[pmeaad3a2dbib6] Chong S P, Merkle C W, Leahy C, Srinivasan V J (2015). Cerebral metabolic rate of oxygen (CMRO_2_) assessed by combined Doppler and spectroscopic OCT. Biomed. Opt. Express.

[pmeaad3a2dbib7] Clanton T L, Hogan M C, Gladden L B (2013). Regulation of cellular gas exchange, oxygen sensing, and metabolic control. Compr. Physiol..

[pmeaad3a2dbib8] Collins J A, Rudenski A, Gibson J, Howard L, O’Driscoll R (2015). Relating oxygen partial pressure, saturation and content: the haemoglobin-oxygen dissociation curve. Breathe (Sheff).

[pmeaad3a2dbib9] Culver J P, Durduran T, Furuya D, Cheung C, Greenberg J H, Yodh A G (2003). Diffuse optical tomography of cerebral blood flow, oxygenation, and metabolism in rat during focal ischemia. J. Cereb. Blood Flow Metab..

[pmeaad3a2dbib10] Dahlqvist M K, Thomsen K J, Postnov D D, Lauritzen M J (2020). Modification of oxygen consumption and blood flow in mouse somatosensory cortex by cell-type-specific neuronal activity. J. Cereb. Blood Flow Metab..

[pmeaad3a2dbib11] Davis T L, Kwong K K, Weisskoff R M, Rosen B R (1998). Calibrated functional MRI: mapping the dynamics of oxidative metabolism. Proc. Natl. Acad. Sci. USA.

[pmeaad3a2dbib12] Dunn A K, Devor A, Dale A M, Boas D A (2005). Spatial extent of oxygen metabolism and hemodynamic changes during functional activation of the rat somatosensory cortex. Neuroimage.

[pmeaad3a2dbib13] Goodwin J A, Vidyasagar R, Balanos G M, Bulte D, Parkes L M (2009). Quantitative fMRI using hyperoxia calibration: reproducibility during a cognitive Stroop task. Neuroimage..

[pmeaad3a2dbib14] Grubb R L, Raichle M E, Eichling J O, Ter-Pogossian M M (1974). The effects of changes in PaCO_2_ on cerebral blood volume, blood flow, and vascular mean transit time. Stroke.

[pmeaad3a2dbib15] Hoge R D, Atkinson J, Gill B, Crelier G R, Marrett S, Pike G B (1999a). Linear coupling between cerebral blood flow and oxygen consumption in activated human cortex. Proc. Natl. Acad. Sci. USA.

[pmeaad3a2dbib16] Hoge R D, Atkinson J, Gill B, Crelier G R, Marrett S, Pike G B (1999b). Investigation of BOLD signal dependence on cerebral blood flow and oxygen consumption: the deoxyhemoglobin dilution model. Magn. Reson. Med..

[pmeaad3a2dbib17] Huppert T J, Allen M S, Benav H, Jones P B, Boas D A (2007). A multicompartment vascular model for inferring baseline and functional changes in cerebral oxygen metabolism and arterial dilation. J. Cereb. Blood Flow Metab..

[pmeaad3a2dbib18] Jones M, Berwick J, Johnston D, Mayhew J (2001). Concurrent optical imaging spectroscopy and laser-Doppler flowmetry: the relationship between blood flow, oxygenation, and volume in rodent barrel cortex. Neuroimage.

[pmeaad3a2dbib19] Jones M, Berwick J, Mayhew J (2002). Changes in blood flow, oxygenation, and volume following extended stimulation of rodent barrel cortex. Neuroimage.

[pmeaad3a2dbib20] Kainerstorfer J M, Sassaroli A, Hallacoglu B, Pierro M L, Fantini S (2014). Practical steps for applying a new dynamic model to near-infrared spectroscopy measurements of hemodynamic oscillations and transient changes: implications for cerebrovascular and functional brain studies. Acad. Radiol..

[pmeaad3a2dbib21] Kastrup A, Kruger G, Glover G H, Moseley M E (1999). Assessment of cerebral oxidative metabolism with breath holding and fMRI. Magn. Reson. Med..

[pmeaad3a2dbib22] Kety S S, Schmidt C F (1948). The effects of altered arterial tensions of carbon dioxide and oxygen on cerebral blood flow and cerebral oxygen consumption of normal young men. J. Clin. Invest..

[pmeaad3a2dbib23] Kim S G (2018). Biophysics of BOLD fMRI investigated with animal models. J. Magn. Reson..

[pmeaad3a2dbib24] Ko T S (2020). Non-invasive optical neuromonitoring of the temperature-dependence of cerebral oxygen metabolism during deep hypothermic cardiopulmonary bypass in neonatal swine. J. Cereb. Blood Flow Metab..

[pmeaad3a2dbib25] Krnjevic K, Randic M, Siesjoe B K (1965). Cortical Co_2_ tension and neuronal excitability. J. Physiol..

[pmeaad3a2dbib26] Lajoie I, Tancredi F B, Hoge R D (2017). The impact of inspired oxygen levels on calibrated fMRI measurements of M, OEF and resting CMRO_2_ using combined hypercapnia and hyperoxia. PLoS One.

[pmeaad3a2dbib27] Lee J (2021). Opposed hemodynamic responses following increased excitation and parvalbumin-based inhibition. J. Cereb. Blood Flow Metab..

[pmeaad3a2dbib28] Lin A J, Ponticorvo A, Durkin A J, Venugopalan V, Choi B, Tromberg B J (2015). Differential pathlength factor informs evoked stimulus response in a mouse model of Alzheimer’s disease. Neurophotonics..

[pmeaad3a2dbib29] Lin P Y (2013). Non-invasive optical measurement of cerebral metabolism and hemodynamics in infants. J. Vis. Exp..

[pmeaad3a2dbib30] Ma Y (2016). Wide-field optical mapping of neural activity and brain haemodynamics: considerations and novel approaches. Philos. Trans. R Soc..

[pmeaad3a2dbib31] Mandeville J B (1999). Evidence of a cerebrovascular postarteriole windkessel with delayed compliance. J. Cereb. Blood Flow Metab..

[pmeaad3a2dbib32] Mayhew J, Johnston D, Berwick J, Jones M, Coffey P, Zheng Y (2000). Spectroscopic analysis of neural activity in brain: increased oxygen consumption following activation of barrel cortex. Neuroimage..

[pmeaad3a2dbib33] Mintun M A, Raichle M E, Martin W R, Herscovitch P (1984). Brain oxygen utilization measured with O-15 radiotracers and positron emission tomography. J. Nucl. Med..

[pmeaad3a2dbib34] Nilsson B, Siesjo B K (1976). A method for determining blood flow and oxygen consumption in the rat brain. Acta. Physiol. Scand..

[pmeaad3a2dbib35] Novack P, Shenkin H A, Bortin L, Goluboff B, Soffe A M (1953). The effects of carbon dioxide inhalation upon the cerebral blood flow and cerebral oxygen consumption in vascular disease. J. Clin. Invest..

[pmeaad3a2dbib36] Piilgaard H, Lauritzen M (2009). Persistent increase in oxygen consumption and impaired neurovascular coupling after spreading depression in rat neocortex. J. Cereb. Blood Flow Metab..

[pmeaad3a2dbib37] Saetra M J, Solbra A V, Devor A, Sakadzic S, Dale A M, Einevoll G T (2020). Spatially resolved estimation of metabolic oxygen consumption from optical measurements in cortex. Neurophotonics.

[pmeaad3a2dbib38] Sheth S A (2004). Columnar specificity of microvascular oxygenation and volume responses: implications for functional brain mapping. J. Neurosci..

[pmeaad3a2dbib39] Takuwa H, Matsuura T, Nishino A, Sakata K, Tajima Y, Ito H (2014). Development of new optical imaging systems of oxygen metabolism and simultaneous measurement in hemodynamic changes using awake mice. J. Neurosci. Methods.

[pmeaad3a2dbib40] Vazquez A L, Fukuda M, Kim S G (2012). Evolution of the dynamic changes in functional cerebral oxidative metabolism from tissue mitochondria to blood oxygen. J. Cereb. Blood Flow Metab..

[pmeaad3a2dbib41] Verdecchia K, Diop M, Lee T Y, St Lawrence K (2013). Quantifying the cerebral metabolic rate of oxygen by combining diffuse correlation spectroscopy and time-resolved near-infrared spectroscopy. J. Biomed. Opt..

[pmeaad3a2dbib42] Yucel M A, Evans K C, Selb J, Huppert T J, Boas D A, Gagnon L (2014). Validation of the hypercapnic calibrated fMRI method using DOT-fMRI fusion imaging. Neuroimage.

[pmeaad3a2dbib43] Zappe A C, Uludag K, Oeltermann A, Ugurbil K, Logothetis N K (2008). The influence of moderate hypercapnia on neural activity in the anesthetized nonhuman primate. Cereb. Cortex..

